# Putting taxes into the diet equation

**DOI:** 10.2471/BLT.16.020416

**Published:** 2016-04-01

## Abstract

Mexico’s soda tax has reduced sales of sugar-sweetened beverages. Time will tell whether the tax helps to reduce obesity prevalence as well. Andréia Azevedo Soares reports.

“In Mexico, you can easily find people drinking *refrescos* (sodas) and eating tacos in the middle of the morning. On nearly every street corner you find street vendors selling sodas and fried foodstuff,” says Mónica García, a university teacher who lives in Mexico City, referring to her fellow Mexicans’ beloved snack.

“People think that a taco without soda is not a real taco,” says Mónica.

Sodas – bottled or canned sweetened soft drinks – have long been part of Mexican culture. The Coca-Cola Company started bottling and selling sodas in the country in 1926. Fourteen years later the first Coca-Cola vending machines were installed in Mexico City and former president, Vicente Fox, was head of Coca-Cola Mexico before taking office in 2000.

“The origins of this entrenchment date back to the 1950s, when Coca-Cola began intense marketing campaigns in Mexico. By the 1970s, sodas were well established as components of daily cultural life,” writes the nutritionist and campaigner Marion Nestle in her book *Soda Politics*, published last year in the United States of America.

García finds the statistics on her compatriots’ soda intake alarming and that is why she welcomes the 1 peso-per-litre (US$ 0.06) tax on these drinks introduced in January 2014.

“People think that a taco without soda is not a real taco.”

Mónica García

The intake of sugar-sweetened beverages has been increasing worldwide. Studies in the last few years show that this increase is a significant factor driving rising rates of obesity and diet-related diseases, such as type 2 diabetes – the theme of this year’s World Health Day on 7 April.

In Mexico, these drinks are a major source of energy for adults and children especially in the many communities that do not have safe tap water.

The age-adjusted prevalence of diabetes in adult Mexicans increased from 10.2% to 10.7% between 2010 and 2014, according to the *Global status report on NCDs 2014*. This prevalence is the highest among the 34 countries in the Organisation for Economic Co-operation and Development. The figures refer to Mexicans with type 2 diabetes (90–95% of total prevalence) as well as those with type 1 diabetes.

More than two years after the Mexican Congress passed legislation levying an excise tax on sugar-sweetened beverages – Mexico’s “soda tax” – a growing body of evidence suggests that pricing policies can influence purchasing patterns and have an impact on dietary behaviour, according to the 2015 World Health Organization (WHO) report, *Using price policies to promote healthier diets*.

A study published in January in the *BMJ* shows that sales of the taxed beverages in Mexico fell by an average of 6% in 2014, and declined by as much as 12% in the last month of the year. In addition, purchases of water and non-taxed beverages increased by about 4% on average.

“Our study shows that taxing sugar-sweetened beverages has a legitimate place as part of a toolkit for the prevention of obesity. Taxation alone will not solve the problem, but can contribute to its prevention and control,” says Dr Juan Rivera Dommarco, co-author of the *BMJ* study and director of the Mexican Research Centre in Nutrition at the National Institute of Public Health.

The soft drinks manufacturers association of Mexico (the Asociación Nacional de Productores de Refrescos y Aguas Carbonatadas) questioned the researchers’ conclusions, arguing that the measure was regressive in that it hit poor households hardest, but failed to make a significant reduction in Mexicans’ average caloric intake.

“Unfortunately, the soda industry in Mexico is winning the battle for public opinion, saying that the tax doesn’t work and that it is regressive,” argues Rivera.

For Rivera, the impact on the poor has to be measured in terms of improving their health. “Our study found that the reduction in soda purchases was greater among the poor (an average 9% reduction), so we expect greater health gains among poor people,” Rivera says. The money raised by Mexico’s soda tax is being reinvested in obesity prevention, for example, in providing drinking water fountains in low-income schools.

“We expect greater health gains among poor people.”

Juan Rivera Dommarco

“The soda tax is part of a comprehensive strategy to reduce obesity and type 2 diabetes,” Rivera says. "The results in terms of a real reduction in obesity and increase in healthy consumption habits will not show immediately.”

Other measures include the regulation of marketing of sodas and unhealthy foodstuffs, Rivera adds.

“The decrease in soda sales in Mexico is good news, but we don’t have conclusive evidence yet on whether this is actually reducing obesity and type 2 diabetes,” says Dr Gojka Roglic, WHO medical officer and the lead author of a global report on diabetes to be released on World Health Day.

“We don’t know yet what people are drinking instead of these sodas,” she explains.

Rivera says that a national survey is planned between 2016 and 2018. “But the effects on weight are probably going to show in five years or more. We estimate that about 400 000 cases of diabetes could be averted by 2050 if Mexico keeps the soda tax.”

Price policies are designed to influence consumers to make healthier choices when buying drinks, for example, by making sodas more expensive than bottled water.

Mexico’s soda tax is paving the way for the whole Americas region. Last year, Barbados levied a 10% excise tax on sugar-sweetened beverages and plans to reinvest the revenue in health. Dominica levied a 10% excise tax on such drinks and chewing gums and Chile levied an 18% value added tax on drinks containing more than 6.25 grams of sugar per 100 ml.

Marion Nestle believes that such taxes are needed because consumer choice is not as free as consumers may think.

“We cannot take marketing out of the equation. Individual choice is very much dependent on marketing,” says Nestle, who is also a food studies and public health professor at New York University. “Companies put millions of dollars into food marketing. You are not supposed to think critically about what you are buying. Marketing is aimed to act below the radar of critical thinking.”

Marketing techniques for energy-dense sodas and foodstuffs have become extremely sophisticated in the last two decades. The food industry is now targeting low- and lower-middle-income countries that have the potential to become highly profitable markets. Children are targets of marketing too, for the same reason, Nestle explains.

An estimated 41 million children globally under five years were obese or overweight in 2014. *The* *Commission on Ending Childhood Obesity *report, released in January, makes recommendations for governments on how to change this. Sugar taxation is one of the commission’s suggested measures, but there are caveats.

“The soda tax is not a silver bullet. Taxation is one of many instruments available to promote healthier diets and should be seen as part of a package of policies aimed at altering consumers’ choices,” says Dr Francesco Branca, WHO Director of Nutrition for Health and Development.

WHO recommends other price policies such as subsidies for, or lower taxation of, healthy food as well as initiatives to encourage people to eat a healthier diet, avoid tobacco and be more physically active.

Finland has taxed sweets and drinks on and off since 1926. Last year the government of the United Kingdom of Great Britain and Northern Ireland rejected a sugar tax proposed by celebrity chef Jamie Oliver and in the United States of America, the city of Berkeley succeeded in introducing a soda tax after similar proposals in other states including New York had failed.

Hungary introduced a tax on a range of foodstuffs and drinks in 2011, including €0.22 per litre (US$ 0.24) on drinks with more than 0.5% sugar content, the revenues from which are reinvested in health.

In the same year, France adopted a tax on sugar- and artificially-sweetened drinks, including fruit beverages and flavoured waters. Its health impact has not been evaluated yet, but a report by Professor Serge Hercberg found that soda sales decreased for the first time in many years in 2012.

The French tax is generating revenues of about €300 million a year since 2012 (US$ 326 million), according to *Using price policies to promote healthier diets*. Co-author, João Breda, programme manager for nutrition, physical activity and obesity in WHO’s Regional Office for Europe, says: “We need to try to find ways to guarantee that the revenue from taxing unhealthy products is reinvested in health-related initiatives.”

**Figure:**
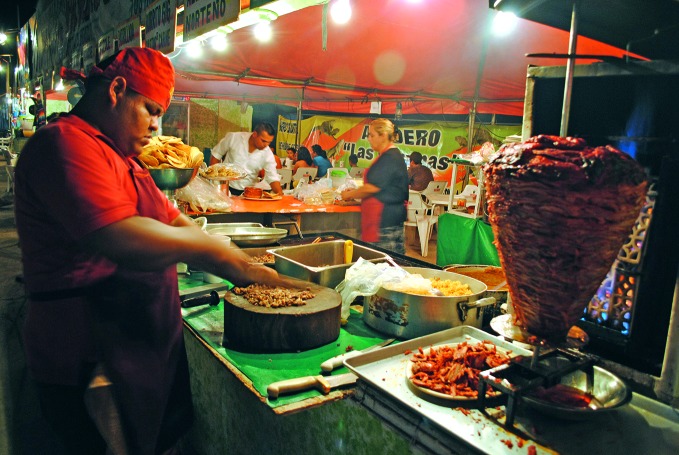
Preparing tacos in Baja California, Mexico

Courtesy of Gabriel Flores Romero/Flickr

**Figure:**
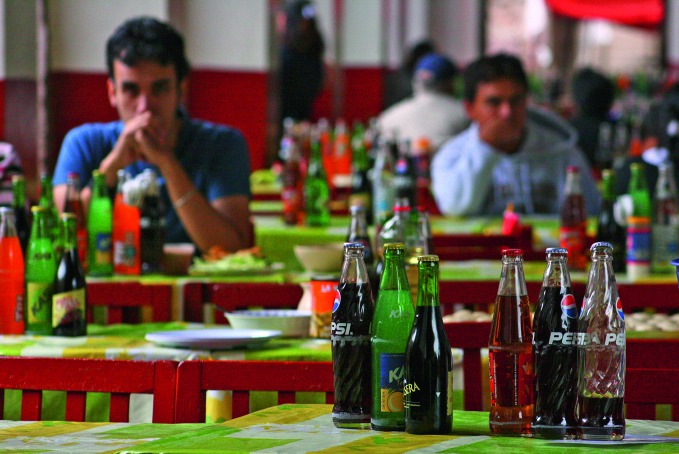
Soda bottles on a table in Morelia, Michoacán, Mexico

Courtesy of Júbilo Haku on Flickr

